# Paradoxical Stress Fracture in a Patient With Multiple Myeloma and Bisphosphonate Use

**DOI:** 10.7759/cureus.9837

**Published:** 2020-08-18

**Authors:** Edwin Chiu, Michael Cabanero, Gurinder Sidhu

**Affiliations:** 1 Medicine, State University of New York (SUNY) Downstate Medical Center, Brooklyn, USA; 2 Anatomic Pathology, University Health Network, Toronto, CAN

**Keywords:** atypical femoral fracture, multiple myeloma, bisphosphonates, stress fracture

## Abstract

Multiple myeloma (MM) is a plasma cell disorder with related organ dysfunction, including hypercalcemia, renal insufficiency, anemia, and bone disease. Osteolytic bone lesions that result in pain and pathologic fractures are a major source of morbidity and the use of bisphosphonates is generally safe and effective treatment in reducing myeloma-related skeletal fractures and associated morbidity. We present a 73-year-old African American woman with MM in remission and on intravenous (IV) bisphosphonate therapy in the past five years who reported gradually worsening bilateral thigh pain of six months duration. A bone survey showed no neoplastic focus, and bilateral hip X-rays showed incomplete insufficiency stress fractures with characteristic features suspicious for bisphosphonate-related atypical femoral fracture (AFF). Increasingly reported in the literature, bilateral AFF is a unique and serious adverse effect for patients on bisphosphonates. Our case illustrates the distinct challenges in managing a patient with MM on long-term bisphosphonate therapy who suffered bilateral atypical femoral fractures, an uncommon presentation of a relatively rare phenomenon. It is important to balance the established benefits of bisphosphonate therapy with potential fracture risk and be particularly vigilant about adverse effect monitoring and timely intervention.

## Introduction

Multiple myeloma (MM) is a terminally differentiated plasma cell disorder that can produce associated organ dysfunction, including hypercalcemia, renal insufficiency, anemia, and bone disease [1]. MM accounts for 1% neoplastic disorders with approximately 15,000 cases diagnosed each year and the median age of onset is 65 years. Of the patients newly diagnosed with MM, 70-80% will have osteolytic lesions related to a skeletal-related event (SRE), including cord compression, bone pain, and pathologic fracture [2]. In general, bisphosphonates (BP) are safe and well-tolerated therapy that reduces the incidence of SRE while improving patients’ quality of life and survival with MM [3-4]. Long-term BP therapy is associated with the serious adverse effect of osteonecrosis of the jaw (ONJ) and atypical femoral fractures [5-7]. Patients with MM are now living longer with antimyeloma therapy and may be especially susceptible to atypical femoral fractures with concomitant factors of advanced age, osteolytic lesions, and need for long-term BP therapy. We describe a case of bilateral atypical femoral fractures in a woman with MM in remission on long-term intravenous BP therapy and review the literature on this patient population.

## Case presentation

The patient was a 73-year-old African American woman with a history of hypertension and MM who presented with gradually worsening bilateral thigh pain (right side worse than the left) of six months duration. She denied previous trauma and associated weakness or numbness. The patient was diagnosed with MM (isotype: immunoglobulin G λ) at age 63 and was initially treated with thalidomide and dexamethasone and switched to bortezomib upon relapse. The patient thereafter has remained in complete remission. Since diagnosis, the patient received intravenous zoledronic acid 4 mg every month for the past five years and on vitamin D and calcium supplementation. Complete blood count (CBC) was remarkable for hemoglobin 10.7 g/dL with mean corpuscular volume (MCV) 81.9 fL. The chemistry panel was unremarkable (Tables 1-2).

**Table 1:** Selected serum laboratory tests and biopsy result WBC: white blood cell; MCH: mean corpuscular hemoglobin; MCHC: mean corpuscular hemoglobin concentration; RDW: red cell distribution width; INR: international normalized ratio; IgG: immunoglobulin G

Serum Laboratory Tests (1/15/2014)	Value	Reference Values
Basic Metabolic Panel		
Sodium	140 mmol/L	136-145 mmol/L
Potassium	3.4 mmol/L	3.5-5.1 mmol/L
Chloride	105 mmol/L	98-107 mmol/L
Bicarbonate	26 mmol/L	21-31 mmol/L
Blood Urea Nitrogen	116 mg/dL	7-25 mg/dL
Creatinine	0.67 mg/dL	0.7-1.3 mg/dL
Calcium	8.4 mg/dL	8.2-10 mg/dL
Alkaline Phosphatase	64 units/L	34 - 104 units/L
Vitamin D 1,25 (OH)2, Total	105.2 pg/mL	10 - 75 pg/mL
Vitamin D, 25-OH, Total	38.6 ug/dL	30 - 95 ug/dL
Parathyroid Hormone, intact	121.3 pg/mL	15 - 65 pg/mL
Complete Blood Count		
WBC	9.44 x 10^3 ^/microL	4.8-10.8 x 10^3 ^/microL
Hemoglobin	10.2g/dL	12-16 g/dL
Hematocrit	31.5%	37-47%
Platelet	521 x 10^3 ^/microL	130-400 x 10^3^/microL
MCV	78.2 fL	81-99 fL
MCH	25.4 pg	27-31 pg
MCHC	32.5 g/dL	33-37 g/dL
RDW	15 %	11.5-14.5%
Neutrophil %	79.1%	40-74%
Lymphocyte %	11.8%	19-48%
Monocyte %	7.7%	3.4-9%
Eosinophil %	0.4%	0-7%
Basophil %	0.1%	0-1.5%
Neutrophil Absolute	7.5 x10^3 ^/microL	1.9-8 x10^3 ^/microL
Lymphocyte Absolute	1.1 x10^3 ^/microL	0.9-5.2 x10^3 ^/microL
Monocyte Absolute	0.7 x10^3 ^/microL	0.2-1 x10^3 ^/microL
Eosinophil Absolute	0.0 x10^3 ^/microL	0-0.8 x10^3 ^/microL
Basophil Absolute	0.0 x10^3 ^/microL	0-0.2 x10^3 ^/microL
Coagulation Profile		
Prothrombin Time	14.8 seconds	10.5-13.1 seconds
INR	1.3	2.0-3.0
Activated Prothrombin Time	27.2 seconds	28.9-38.3 seconds

Serum Protein Electrophoresis		
Total Protein	6.9 g/dL	6-8.3 g/dL
Albumin %	47.9 %	52-65%
Alpha 1%	3.3 %	2.5-5%
Alpha 2%	14.0 %	7-13%
Gamma %	22.2 %	12-22%
Albumin E Absolute	3.3 g/dL	3.2-5.6 g/dL
Alpha 1 Absolute	0.23 g/dL	0.1-0.4 g/dL
Alpha 2 Absolute	0.97 g/dL	0.4-1.2 g/dL
Beta Absolute	0.86	0.5-1.1
Gamma Absolute	1.53	0.5-1.6
Interpretation: monoclonal gammopathy; immunofixation shows a monoclonal IgG lambda band		
January 1, 2014, Intramedullary Reamings, Left Leg Bone Biopsy: Plasma cells (CD138+) account for <5% cells overall but are present in rare aggregates of 10-15 cells. The vast majority of cells are labmda+, giving a kappa:lambda ratio of <1:10. Normal hematopoiesis noted with a 3:1 myeloid:erythroid ratio and adequate megakaryocytes.		

**Table 2:** Serum-free light chains

9/17/2013 Serum-Free Light Chains		
Free Kappa, serum	9.24	3.3-19.4 mg/L
Free Lambda, serum	121.4	5.71-26.3 mg/L
Free Kappa/Lambda ratio	0.08	0.26-1.65

**Figure 1:**
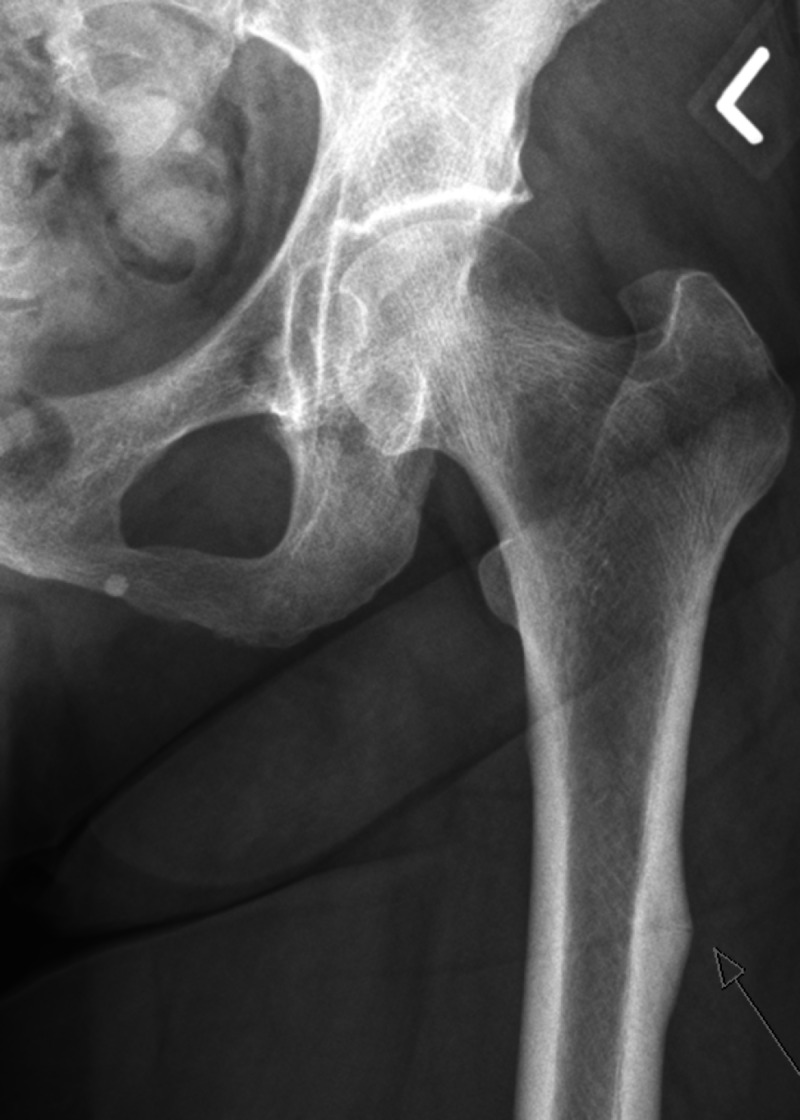
X-ray left hip shows a left atypical femoral fracture (AFF) in the proximal femur The cortical periosteal thickening is adjacent to the faint translucent fracture that is extending from the lateral cortex into the medulla.

**Figure 2:**
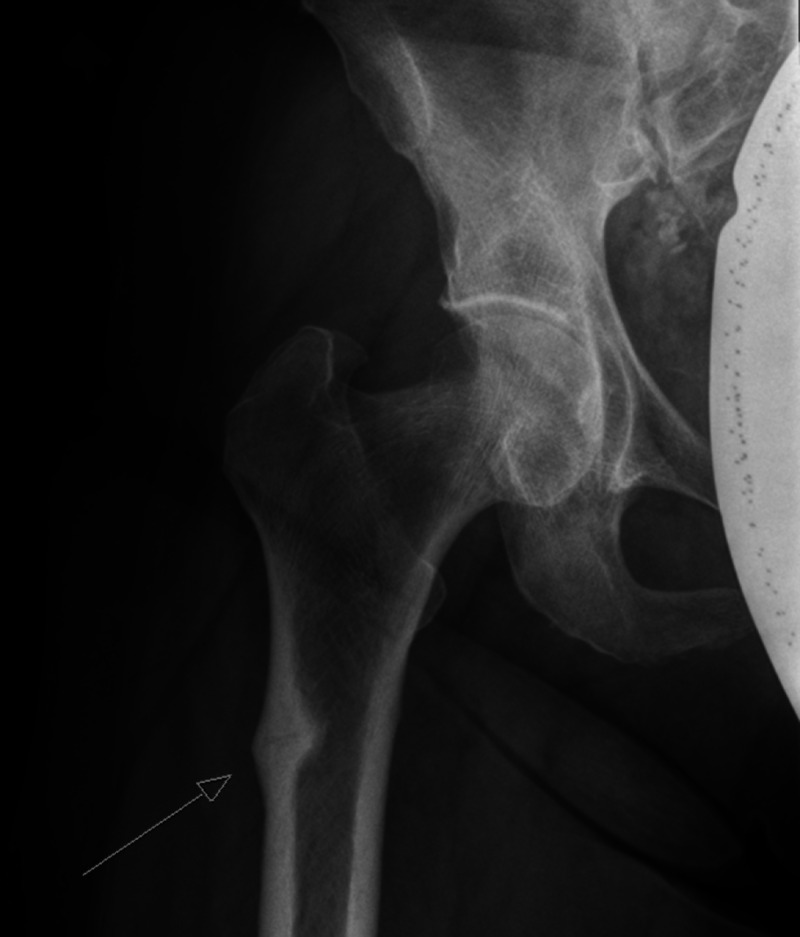
X-ray right hip shows a right atypical femoral fracture The lateral cortical periosteal thickening and oblique fracture is more pronounced.

**Figure 3:**
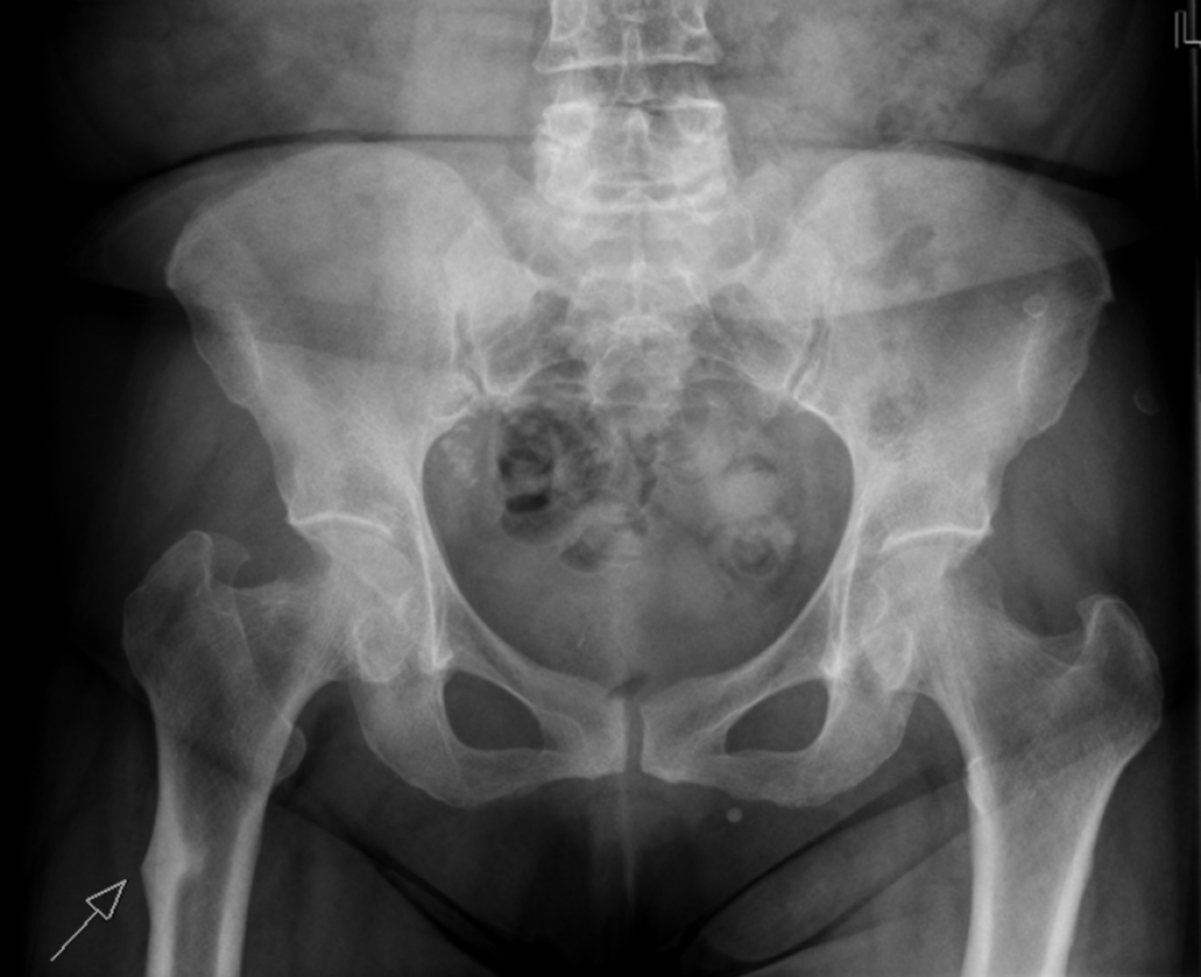
Pelvic X-ray shows bilateral atypical femoral fractures

The intact parathyroid hormone was mildly elevated at 121.3 pg/mL. Vitamin D and calcium levels were normal. Prior bone densitometry one year earlier showed osteopenia of lumbar spine T score -1.5, osteoporosis of left forearm T score -2.9, and left femoral neck T score -0.8. Bilateral hip X-rays showed bilateral incomplete insufficiency stress fractures along the lateral aspect of both proximal femoral diaphyses (Figures 1-3).

**Figure 4:**
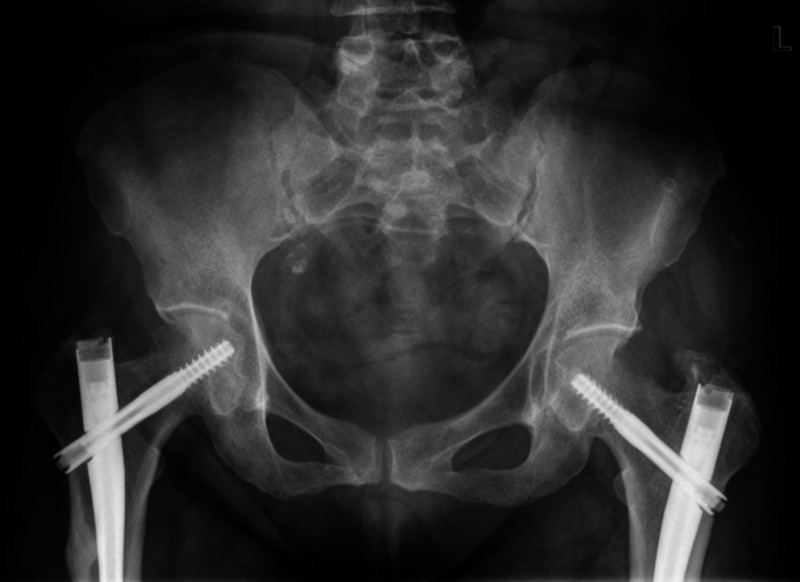
Pelvic X-ray shows intramedullary nail fixation of bilateral atypical femoral fractures

The subsequent bone survey corroborated these findings and did not identify any other neoplastic focus. The characteristic location and presentation were suspicious for bisphosphonate-related atypical femoral fracture. Zoledronic acid was discontinued, and the patient was admitted for prophylactic intramedullary nailing of bilateral proximal femurs (Figure 4).

**Figure 5:**
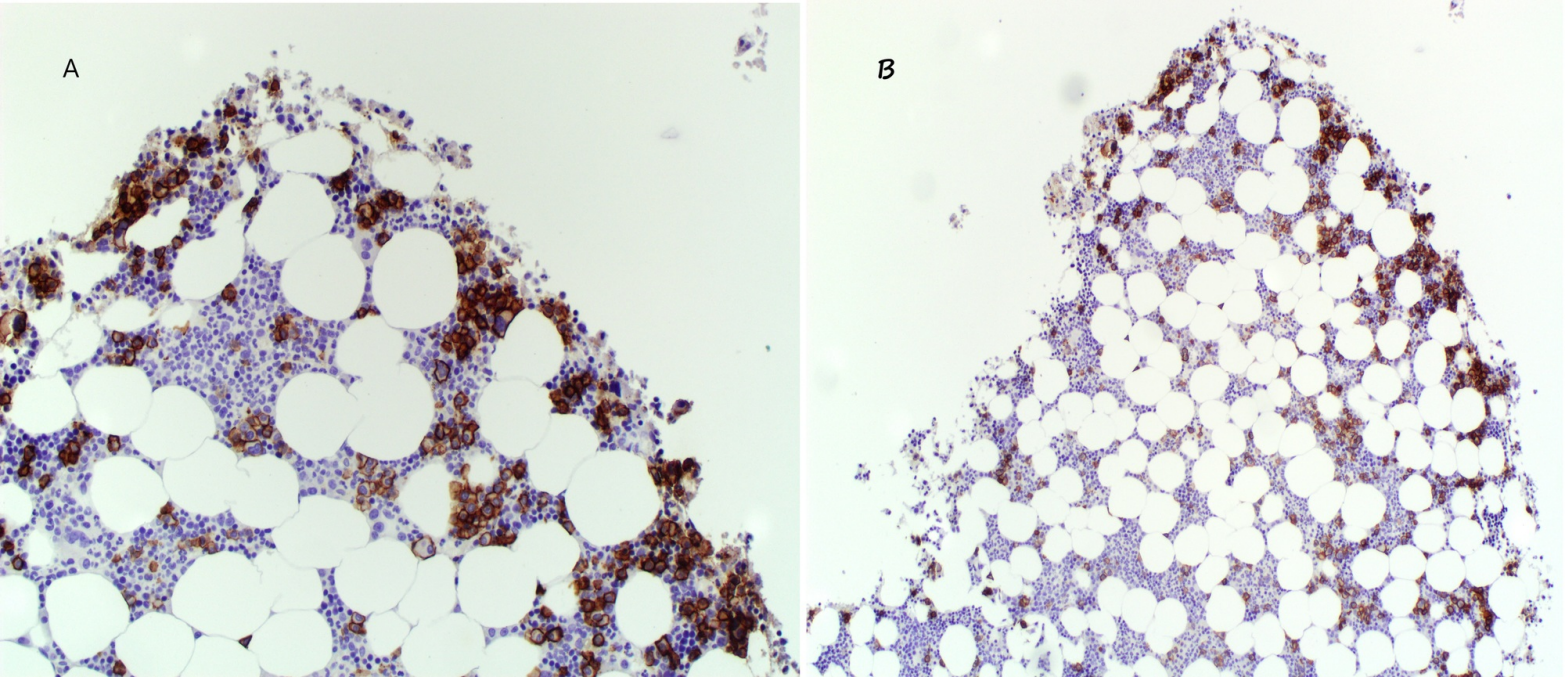
Monotypic plasmacytosis in bone biopsy obtained during perioperative intramedullary nailing Plasma cells with CD138+ (immunohistochemistry marker for plasma cells) account for less than 5% cell aggregates in the overall specimen (5B) with immunoglobulin G λ predominance. Overstaining is present. An enlarged view is shown in 5A.

Biopsy of the lesion at the time of intramedullary nailing showed monotypic plasmacytosis in fragments of bone with <5% plasma cells (Figure 5) and M-protein 1.5 grams initially suggestive of relapse. However, a review of prior bone marrow histopathology before antimyeloma treatment demonstrated less than 30% plasma cell involvement in the bone marrow and with treatment, the patient remained in clinical remission. Given that the pathologic and radiologic features were inconsistent with myeloma osteolytic fracture and overall clinical context, the patient likely suffered AFF secondary to BP use. The patient then underwent physical and occupational therapy with good recovery.

## Discussion

Multiple myeloma (MM) bone destruction is well-characterized by several pathways in the altered bone-marrow microenvironment. Malignant myeloma cells secrete potent factors, such as interleukin-3 (IL-3), macrophage inflammatory protein-1α (MIP-1α), receptor activation of NFκB ligand (RANKL), and tumor necrosis factor (TNF-α), to increase the number and activity of osteoclast-mediated bone destruction. This process releases growth factors required for tumor growth, colonization, and metastatic dissemination [8]. Through a wingless-type signaling pathway inhibitor, myeloma cells also impair osteoblast differentiation [1].

Bisphosphonates (BP) work on these pathways to reduce myeloma skeletal-related effects by inhibiting osteoclast-mediated bone resorption, inducing osteoclast apoptosis, and accumulating metabolites toxic to myeloma cells. BP with high osteoclast affinity concentrates in areas of high bone remodeling and distributes via chemical desorption. Other mechanisms suggest that BP reduce tumor-associated angiogenesis and exhibit anticancer activity by activating immune surveillance [8-9]. The cumulative effect is that BP with antimyeloma therapy reduce myeloma-related morbidity and mortality [3-4]. BP, however, is not without adverse effects. Long-term use has been associated with osteonecrosis of the jaw (ONJ), a well-known serious event that involves necrosis or osteomyelitis of the jaw with an estimated incidence of 1%-11% depending on BP type and comorbidities [10]. There are documented reports of atypical femoral fracture (AFF) in the context of bisphosphonate use, often involving the subtrochanteric and femoral shaft in a transverse or oblique manner (<30 degrees) with minimal or no trauma. Associated features include prodromal thigh or groin pain and characteristic radiologic signs (i.e. cortical “beak”) [7,10-14]. The estimated incidence of AFF in the general population on BP ranges from 3.2 to 50 cases per 100,000 person-years [15]. The true incidence of AFF in patients with MM on BP is unknown given that data are from reported retrospective case series [5-7,10-12]. There may also be confounding factors with patients with MM, including osteolytic lesion involvement, advanced age, comorbidities, and concomitant immunomodulating treatment.

Although the exact pathogenesis of AFF remains unclear, there are several proposed theories on the AFF fracture mechanism and BP effect on bone modeling suppression. Bone fractures occur when the repetitive load exceeds the bone’s ability for self-repair. These can be classified as stress fractures (abnormal load on normal bone) or insufficiency fractures (normal load on abnormal bone). Over time, these bone insults or “microcracks” in the setting of BP-inhibited repair coalesce into a larger defect that forms the AFF [15-17]. Moreover, the long half-lives of BP could lead to the oversuppression of bone remodeling processes and potentially contribute to AFF development [18]. This was suggested by Odvina et al., where patients on BP over three to eight years with atypical fractures had histomorphometric evidence of suppressed bone turnover and reduced generalized bone formation, particularly in intracortical and endocortical bone surfaces [17]. After an incident fracture, patients on BP showed evidence of delayed fracture healing while patients off BP healed satisfactorily. In animal studies, bone biopsies from BP-treated animals demonstrate microdamage accumulation and reduced biomechanical bone properties despite the appearance of thickened cortices, suggesting a compromised skeletal structure [18]. Although data is limited on the duration of BP therapy and the development of AFF, there is strong statistical and biological plausibility to suggest BP dose and time dependence [15,17].

The initial radiologic clue to an AFF would be the development of an early focal or generalized periosteal thickening (“cortical spike”) that is adjacent to the evolving fracture, which can be either transverse or oblique (< 30°) in the femur. Fracture location spans the proximal femoral shaft up to the supracondylar region and is rarely comminuted. AFF are diagnosed by these features and by the exclusion of trauma, pathologic fracture associated with a malignant lesion, and prosthetic-related fracture [16]. Once detected, these lesions should be further evaluated by magnetic resonance imaging (MRI) or computed tomography (CT) to better assess bone integrity before fractures clinically manifest. MRI can assess the degree of bone marrow plasma cell infiltration and response to chemotherapy and can detect increased cortical signaling, an early feature in developing cortical thickening and future AFF site development. CT can evaluate the stability of AFF lesions and is useful in planning biopsy and surgical intervention. The disadvantages of these modalities are cost, prolonged image acquisition time, and patient tolerance [19]. Once AFF occurs, a patient’s bone parameters and symptoms should be assessed and appropriate nutritional supplementation of vitamin D and calcium be given. BP should be immediately discontinued and the patient’s bone status should be monitored for radiologic stabilization [15,17]. Asymptomatic patients should have interval monitoring as guided by clinical judgment. Symptomatic patients, depending on severity, may be conservatively treated with analgesics for 2-3 months and should be considered for prophylactic nail fixation of involved bone [16]. Several consensus statements have considered the discontinuation of intravenous BP therapy after two years if patients with MM remain stable; however, these concerns were of ONJ and not of AFF [2,4]. No firm recommendation can be made on when to restart bisphosphonate therapy after discontinuation.

Our case of a patient with multiple myeloma who suffered bilateral AFF on long-term intravenous BP treatment (>5 years) is an uncommon presentation for a relatively rare phenomenon. A review of similar cases shows that most patients with similar prodromal pain and radiologic findings present with unilateral AFF and rarely with bilateral femoral fractures (Table 3).

**Table 3:** Published reports of patients with multiple myeloma and atypical femoral fractures on bisphosphonates MM = Multiple Myeloma, STF = Subtrochanteric, AFF = Atypical Femoral Fracture, F = Female, M = Male, -- denotes no data * in this series, the two patients with MM had bilateral subtrochanteric fractures, however, data on age, sex, treatment, and symptoms were not individually reported.

	Study	Patients	Sex	Age	Fracture type and location	Biphosphonate duration (years)	Steroid use	Prodromal symptoms	Bilateral fractures
1	Ward et al. [7]	N=1	M	79	Right STF	Zoledronic acid/pamidronate (9+ years)	--	Hip/thigh pain	No
2	Napoli et al. [11]	N=1	F	56	Left AFF	Pamidronate (2 years); Zolendronic acid (4 years)	Prednisone 6mg	--	No
3	Chang et al. [12]	N=2/23	14 F; 9 M	73.1+ 10.8	Bilateral AFF*	Pamidronate/zoledronic acid (median 1.9 years)	--	--	2 patients
4	Puhaindran et al. [14]	N=1	F	64	Right STF	pamidronate (3.4 years); zoledronic acid (2.5 years)	--	Thigh pain	yes
5	Wernecke et al. [13]	N=1	F	72	Bilateral STF	Zoledronic acid (6 years); pamidronate (5 years)	--	Thigh pain	yes
6	Grasko et al. [10]	N=1	M	57	Left STF	Pamidronate (7 years); zoledronic acid (3 years)	Yes	Thigh pain	no

There are several limitations to our case report. Our experience is limited to one retrospective case, however, our patient presents with classic symptoms and signs of AFF consistent with the others’ experiences [5-7,10-14]. Our patient has several high-risk factors that could confound AFF development, including advanced age, generalized osteopenia, comorbidities, and antimyeloma therapy. The overall clinical picture did not support osteolytic fractures as evidenced by the limited bone marrow involvement of plasma cells on biopsy during intraoperative medullary fixation and radiographic features. A biopsy sampling error was considered, however, other reports of AFF with submitted intraoperative bone specimens during the repair of pathologic fractures while on BP therapy also documented no evidence of malignancy-related fractures [12]. MRI and CT could have been helpful, however, they were not performed, as X-rays of the bilateral femurs and clinical history were sufficient for diagnosis. In cases of ambiguity, MRI, CT, or a higher imaging modality would be pursued. Despite the abovementioned limitations, our case demonstrates an important lesson.

## Conclusions

Clinicians seeing patients with MM and other high-risk features must be vigilant of AFF development when starting BP therapy and monitoring a patient’s bone status. Patients can be clinically monitored by simple screening inquiries on prodromal symptoms (i.e. thigh pain and ambulation) and interval radiologic imaging as routinely indicated. More research remains to be done to understand this serious adverse effect. In this vulnerable patient population, the challenge is to appropriately balance the benefits of bisphosphonate therapy while minimizing the risks of fracture.
